# Controlled drug release for inflammatory bowel disease (IBD)

**DOI:** 10.6026/97320630016513

**Published:** 2020-07-31

**Authors:** D Asha, S Jeganath, G Bupesh, UK Sahoo, M Bhaskar, K Pandian

**Affiliations:** 1 Department of Microbiology, Saastra College of Pharmaceutical Education and Research, Nellore, AP, India; 2 Department of Pharmaceutics, School of Pharmaceutical Sciences, Vels Institute of Science, Technology And Advanced Studies (VISTAS), Pallavaram, Chennai-600117, India; 3 Research and Development Wing, Central Research Laboratory, Sree Balaji Medical College and Hospital (SBMCH), BIHER, Chennai-600044, India; 4 Department of Forestry, Mizoram University, Aizawl, Mizoram -796004; 5 Department of Forest Science, School of Science, Nagaland University (Central), Lumami, Zenheboto, Nagaland-798627, India; 6 Department of Inorganic Chemistry, University Madras, Chennai, India

**Keywords:** Budesonide Crohn's disease, lag time, crospovidone, 3^2^factorial designs

## Abstract

Controlled drug release in formulation is an important area of research. Formulations using crospovidone as super-disintegrants to achieve immediate release once it reaches the ileo- cecal region is relevant. The Eudragit L30D pH dependent polymer that allows drug release after a lag time of 4-5 hrs to achieve desired drug release from the drug delivery system is critical. Hence, pre-formulation to study drug-polymer interaction is essential. The linear correlation between the predicted and actual values for all the batches of optimization is shown with high correlation coefficient (r-value). Therefore, the designed formulation is promising for ileo-cecal targeted pulsatile drug delivery system in the management of Crohn's disease.

## Background

Inflammatory bowel disease (IBD) is a group of inflammatory conditions of the digestive system [1]. IBD is considered as chronic, incurable disease. IBD occurs in variety of forms; they are Crohn's disease (CD) and Ulcerative colitis (UC). Currently available drugs for the treatment of IBD include anti-inflammatory agents such as mesalazine, corticosteroids (prednisolone, methyl prednisolone and budesonide), immunosuppressive agents (azathioprine and cyclosporine), antibiotics (ciprofloxacin, metronidazole) and monoclonal antibodies (Infliximab) to reduce mucosal inflammation [2,3]. While these treatments are effective and gives symptomatic relief to the patients with innumerable side effects [4]. Interest on targeted delivery system for the treatment of Ulcerative colitis (UC), Crohn's disease (CD and some bowel cancers are increasing. This requires the local delivery of drugs with minimal side effects. A therapeutic agent protected from degradation will be released and/or absorbed in the upper GIT of the proximal colon. Corticosteroid budesonide acts as a mainstay drug for treating moderate to severe IBD [5]. Budesonide has low incidence of adverse effects and high topical effects and is an important drug in the pharmacotherapy of IBD [4]. A recent study on budesonide for the treatment of IBD have found that budesonide is extensively and presystemically metabolized in the intestinal wall and the liver. The bioavailability is only 10-15 % irrespective of its route of administration [6,7]. Hence the colonic delivery of budesonide needs to be optimized by a more reliable targeted system. Therefore, it is of interest to develop colon-targeted delivery of budesonide coated with pH sensitive polymer to improve the delivery of drug at the site of action [8].

## Materials and Methods:

Budesonide was purchased from Zydus Cadila Pvt. Ltd. (India) Eudragit L30D was provided by the Research-Lab Fine Chem. Industries (Mumbai, India). Crospovidone, Polyethylene Glycol (PEG400) and PVPK30 were purchased from Research-Lab Fine Chem. Industries (Mumbai, India). All chemicals used in this study were of analytical grade.

### Preformulation study:

Confirmation of Budesonide drug was completed using melting point determination [3], UV [4] (JASCO V630, Japan), FTIR (JASCO IR 4100) and DSC (Mettler Toledo Stare DSC 822c, Germany). The observed value was found complying with the reported standard value [9, 10].

### Preparation of Budesonide pulsatile release tablets:

The granules were prepared by wet granulation method. The drug Budesonide, crospovidone and lactose were passed through sieve 40# separately and blended thoroughly. After proper mixing then slowly added the binding solution containing PVP K-30 in IPA till fine uniform granules were obtained. The wet mass was passed through sieve 16# and dried at 50°C for 30 minutes to get the moisture content less than one. The dried granules was then lubricated with magnesium stearate passed through sieve 40#. The lubricated granules were compressed on cad mach tablet punch machine for all formulations [11,12]. Granules were evaluated for micrometric properties such as bulk density, tapped density, angle of repose and Hausner ratio.

### Coating of Eudragit L30D over drug containing tablets:

Eudragit L30D coating dispersion requires addition of polyethylene glycol as plasticizer and stirred the solution for few minutes with a magnetic stirrer [13]. This solution was sprayed over the above processed tablets up to 5, 10, 15, 20, 25, 30 and 35% weight gain.

### Statistical optimization of budesonide formulation using 32 factorial designs:

The data obtained from the dissolution profile of the preliminary experimental batches [14] and variables with range of concentrations were selected for a 32 randomized full factorial design. In this design two factors were evaluated, each at three levels and experimental trials were performed at all 9 possible combination.

Full factorial design were carried out using 2 factors namely extent of Eudragit L30D (% w/w) coating weight gain and the extent of crospovidone (% w/w) coating weight gain as independent variables (Table 1 and Table 2). The optimization study was performed using the Design Expert® software (Design Expert trial version 9; State-Ease Inc., Minneapolis, MN, USA).

Polynomial models including interaction and linear terms were generated for the entire response variables using multiple linear regression analysis (MLRA) approach. The general form of the MLRA model is represented in the following equation:Y = b_0_ + b_1_X^1^+ b_2_X^2^ + b_12_X^1^X^2^+ b_11_X^12^+ b_22_X^22^.

In above equation, Y is the dependent variable; b_0_ is the arithmetic average of all the quantitative outcomes of nine runs. b_1_, b_2_, b_12_, b_11_, b_22_ are the estimated coefficients computed from the observed experimental response values of Y and X_1_ and X_2_ are the coded levels of the independent variables. The interaction term (X_1_X_2_) shows how the response values change when two factors are simultaneously changed. The polynomial terms (X^12^,X^22^) are included to investigate nonlinearity [15] Statistical validity of the polynomials was established on the basis of analysis of variance (ANOVA) provision in the software. Level of significance was considered at p < 0.05.The best-fitting mathematical model was selected based on the comparison of several statistical parameters, including the coefficient of variation (CV), the multiple correlation coefficient (R^2^), the adjusted multiple correlation coefficient (adjusted R^2^) and the predicted residual sum of squares (PRESS) provided by the software. PRESS indicates how well the model fits the data and for the chosen model, it should be small relative to the other models under consideration. The software also generated the 3D response surface graphs and the 2D contour plots. These plots are very useful to understand interactive effects of the factors on responses.

### Evaluation parameters of optimized formulations:

The parameters such as flow ability of granules (by calculating angle of repose, Bulk density, Tapped density, Hausner's ratio, Tablet thickness and diameter, Hardness of tablet, Friability test, Weight variation and Drug content uniformity were evaluated for optimized prepared formulations. Flow ability of granules was determined by calculating angle of repose by funnel method [11]. Bulk density was determined by placing optimized tablet granules into graduated cylinder andmeasuring the volume and weight. Tapped density was determined with the help of tapped density tester apparatus. Hausner's ratio provides an indication of the degree of densification, which could result from vibration of the feed hopper. Hausner's ratio closer of less than 1.25 indicates good flow, while greater than 1.5 indicates poor flow materials. Tablet thickness and diameter were accurately measured by using digital Vernier caliper in mm. Results were expressed as mean values ± standard deviations (SD). Hardness of tablet was determined using the Monsanto hardness tester. Friability test was done by Roche friabilator. Twenty tablets were selected at random and average weight was determined. Then individual tablets were compared with the average weight [13]. For determination of drug content, weighed and powder 5 tablets, then weighed accurately a quantity of the powder equivalent to 9mg of budesonide were transferred to the conical flask and suitably diluted with 10mL phosphate buffer (pH 7.4) respectively. The solution was filtered through Whatmann filter paper (no.41), and assayed at 245nm, using a JASCO V630, Japan UV- spectrophotometer.

## Results:

### Pre-formulation study:

The melting point of budesonide was determined by using capillary method and was found to be 241-245°C (Standard 245-255°C) which complies with the reported value.

### Assessment of the drug-polymer interaction using FTIR:

**Figure 1:**
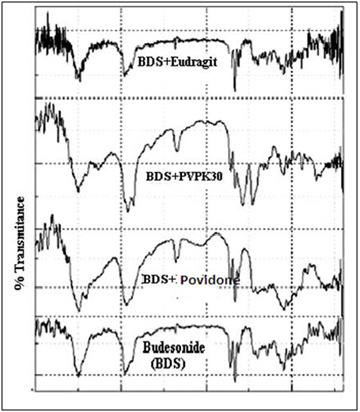
IR spectrums of drug: polymer and physical mixture of Budesonide with crospovidone, PVP K30 and Eudragit L30D

An IR spectrum of budesonide drug sample was observed and compared with the standard spectra. The IR spectra showed no evidence of the chemical interaction between the Budesonide and excipients like, crospovidone, PVP K30, and Eudragit L30D polymers (Figure 1).

### Assessment of the drug-polymer interaction using DSC:

**Figure 2:**
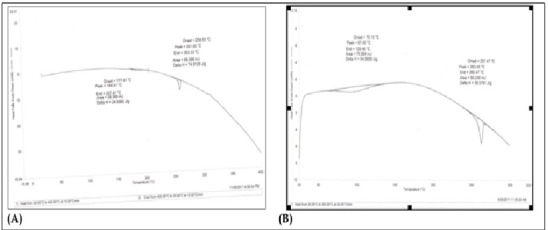
DSC thermo grams of (A) pure Budesonide and (B) drug: polymer physical mixture

The DSC thermo gram for Budesonide shows a sharp melting endothermic peak at 261°C and end at 263.33°C with onset at 258.83°C (Figure 2a). While the endothermic peak of drug polymer mixture was observed at 262.45°C and end at 265.47°C with onset at 257.47°C (Figure 2b) where most of peaks are retained in drug: polymer physical mixture as observed in budesonide pure drug.

### In vitro drug release study of Budesonide experimental trial batches (F1-F15):

In vitro drug release study was conducted at pH 1.2, 7.4 and 6.8 simulated to stomach, small intestine and colon respectively. The formulations S1 to S15showed maximum drug release of about (F1-77.60% at 60 mts, F2-84.88% at 120 mts, F3-91.74% at 180mts, F4-93.87%at 210mts, F5-94.38%at 270mts, F6-93.79%at 240mts, F7-93.28%at 360mts, F8-89.47%at 390 mts, F9-94.47% at 300mts, F10-92.68%at330mts, F11-93.28%at360mts, F12- 91.74%at330mts, F13-94.38%at 360mts, F14-93.79%at 360 mts, F15-93.28%at 390 mts respectively)

### Statistical optimization of formulation using 32 factorial designs:

Based on the results of experimental trial batches, the formulation F11 showed burst release with desirable lag time and hence it was selected for factorial studies to optimize effect of variables on formulation. Further studies with 3^2^ general factorial designs using extent of crospovidone and Eudragit L30D is coating weight gain as variable factors. Nine formulations were generated by the software and coded as OF1-OF9.

### Granules evaluation:

The physical characteristics of the granules (OF1-OF9) such as bulk density, tapped density, Carr's index, Hausner's ratio, angle of repose were determined and the results were tabulated (Table 3).

The bulk densities were ranged from 0.707-0.824 gm/ml. The tapped densities were ranged from 0.830-0.952 gm. /ml. The Carr's compressibility index was ranged from 7.42-15.90%. The Hausner's rations were found to be in the limit 1.08-1.20. The angles of repose of all formulation were found to be between the limit 22.35°-25.58°. All the formulation shows excellent flow properties. So, the granule passes the evaluated tests and subjected to next stage of work compression.

### Tablet thickness and diameter:

The thickness of the tablets was ranged from 3.58-3.77 mm respectively. The diameter of the tablet was shown in between 5.99-6.03 mm. There is no variation in tablet thickness and diameter between the formulations.

### Hardness, friability and weight uniformity of tablets:

The hardness of the tablets was within the range and optimum for controlled release, and ranging from 7.5-8.2 Kg/cm2 for all formulations. The friability of all formulations was ranging from 0.199-0.209 % w/w and passes as per IP limit should not be more than 1 % w/w. The weight uniformity of tablet in all formulation was observed to be within the IP limit 10 %. All formulations were complying with the official test. The values were mentioned in (Table 4).

### In vitro drug release study of optimization batches OF1-OF9:

**Figure 3:**
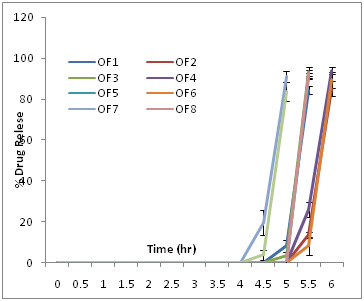
In vitro drug release profile of Budesonide optimization batches OF1-OF9

In vitro drug release study of optimization batches was conducted in pH 1.2, 7.4 and 6.8 simulated to stomach, small intestine and colon respectively. The graphical representation was given in (Figure 3).

### In vitro drugrelease kinetics

To understand the mechanism of drug release from the formulations, the data were treated with zero order (cumulative percent of drug release vs. time), first order (log cumulative percentage of drug remaining vs. time), Higuchi model (cumulative percent of drug release vs square root of time) and Korsmeyer and Peppas (log cumulative percent of drug release vs log time) equations. When the result was plotted according to the zero order equation, the formulations showed good linearity, when the same data was plotted according to the first order equation, Higuchi's equation and Korsmeyer and Peppas equation it shown a fair linearity. The results are given in the (Table 5)which indicates that the release of drug from the formulations follows zero order release kinetic model.

### Statistical analysis of data:

32 full factorial designs were constructed to study the effect of the extent crospovidone (A) and Eudragit L30D coating weight gain (B) on the drug release from the tablets. The layout of the two dependent variables chosen were selected i.e. % cumulative drug released till lag time of 5h and 90% of drug release within 90 minutes after lag time. The experimental runs and observed results were compiled and are shown in (Table 6).

### Full and reduced model assessment of mathematical relationships between dependent and independent variables:

In order to determine the levels of factors, which yield optimum dissolution responses, mathematical relationships were generated between the dependent and independent variables. Full model equation for 90% drug release and lag timeresponses are given below:

### Final Equation in Terms of Coded Factors:

Drug release = +93.52 -1.93 * A -1.39 * B +1.06 * AB -1.09 * A2 -5.12 * B2

### Final Equation in Terms of Actual Factors:

Drug release = -15.82889 +0.28950 *Crospovidone +9.10467 *Eudragit -0.042500 *Crospovidone *Eudragit -0.043467 *Crospovidone^2^ -0.20467 *Eudragit^2^

### Final Equation in Terms of Coded Factors:

Lag time = +4.67 -0.33 * A +0.25 * B 

### Final Equation in Terms of Actual Factors:

Lag time = +4.75000 -0.066667 *Crospovidone +0.050000 *Eudragit 

Coefficients with more than one factor represent interaction between factors while coefficients with quadratic nature and linear model for 90% drug release and lag time respectively. Statistical validation of the polynomial equations generated by Design Expert and estimation of significance of the models was established on the basis of ANOVA provision of the software. ANOVA indicated that assumed regression models were significant and valid for each considered response (Table 7 and Table 8).

The Model F-value of 1.42implied models was significant. There was only a 0.041% chance that a "Model F-Value" this large could occur due to noise. Values of "Prob > F" less than 0.0500 indicate model terms are significant. In this case A and A2 are significant model terms. Values greater than 0.1000 indicate that the model terms are not significant. If there are many insignificant model terms (not counting those required to support hierarchy), model reduction may improve the model.

The Model F-value of 6.82 implied models was significant. There was only a 0.02% chance that a "Model F-Value" this large could occur due to noise. Values of "Prob> F" less than 0.0500 indicate model terms are significant. In this case A and B are significant model terms. Values greater than 0.1000 indicate the model terms are not significant. If there are many insignificant model terms (not counting those required to support hierarchy), model reduction may improve the model.

**Figure 4:**
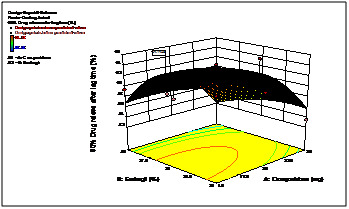
3D response curves of 90% drug release after lag time

**Figure 5:**
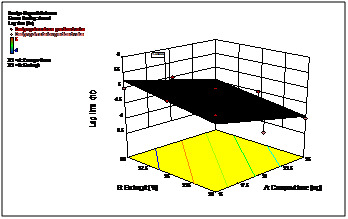
3D response curves of lag time (hr)

The 3D response curves were drawn to estimate the effects of the independent variables on each response, shows the effect of two formulation factors on lag time of 5h. This figure indicates that increase in coating weight gain of Eudragit L30D rises lag time significantly. (Figure 4 and Figure 5) shows the effect of two formulation factors on percent of drug release within 90 min. after lag time of 5h at pH 6.8. This figure confirms that increasing coating weight gain of crospovidone creats more pressure over outer Eudragit L30D coat due to swelling and thus helps in releasing of drug by rupturing or disintegrating the outer membrane.

**Figure 6:**
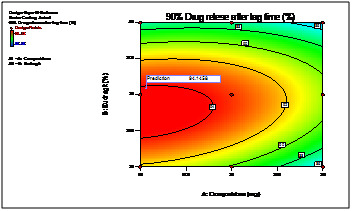
2D contour plot of 90% drug release after lag time

**Figure 7:**
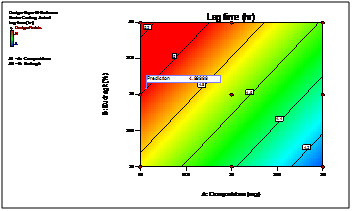
2D contour plot of lag time (hr)

From the 2D contour plots the best area for formulation to obtain desired responses was found (Figure 6 and Figure 7). The best conditions to optimize drug release corresponded to 15.29 mg crospovidone and 25.39% Eudragit L30D weight gain. In order to check the validity of the optimization procedure, a new batch with the predicted levels was prepared.

### Validation of optimum formulations:

A numerical optimization technique by the desirability approach was used to generate the optimum selection of the formulation. The process was optimized for the dependent variables 90% drug release after lag time and lag time. The optimum formulation was selected based on the criteria of attaining the maximum value of % drug release and lag time minimum 5 hr. The predicted and actual values of the optimization batches given by the Design expert software are shown in (Table 9).To justify the validity of the equations, values of X1 and X2 were substituted in equation 2 and 4 to obtain the predicted values of Y1 and Y2. The predicted and observed values were found to be in good agreement.

**Figure 8:**
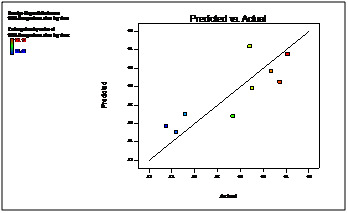
Linearity plot between Predicted and Actual values for 90% drug release after lag time

**Figure 9:**
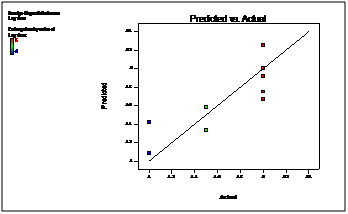
Linearity plot between Predicted and Actual values for lag time (hr)

The linear correlation plots drawn between the predicted and actual values for all the batches of optimization shown in (Figure 8 and Figure 9), which demonstrated high values of R2 0.989 and 0.993 for 90% drug release after lag time and lag time respectively. Thus the low magnitudes of error as well as the values of R2 in the present investigation prove the high prognostic ability of the optimization technique by factorial design.

## Discussion:

Recently the concept of multiparticulate rupturable drug delivery systems has gained significant attention for the local and systemic availability of drugs. IBD including IBS, UC and CD are considered serious colonic disorders. UC, if not treated leads to colon cancer. Currently in the Indian market very few site-specific formulations are available on these disease conditions. CD occurs to any part of GIT but the most susceptible part is "Ileocecal region". For effective treatment of this disease the drug must be release at ileocecal region immediately within lag time is essential. Hence, present study is an attempt to develop, optimize and evaluate of ileocecal targeting rupturable drug delivery system that will release specifically and rapidly in ileocecal region without being released in the upper GIT.

The FT-IR spectrum of drug and polymer mixture reveals that there are no observable characteristic absorption bands. This result strongly implies that the drug is firmly incorporated in polymer matrix during the formulation of tablets.

Thermal analysis is a usual method for the analysis of drugs and excipients. DSC provides idea about melting behavior, purity heat of fusion, pseudo-polymorphism, polymorphism, crystallization, glass transition, and compatibility and chemical reactions of drugs with excipients such. The presence of any impurity in a material shortens its melting point and broadens its melting range by an amount ΔT. According to 32 general factorial designs nine formulation batches were generated by the software and coded as OF1-OF9. All nine batches are evaluated for micromeritic study, in vitro drug release and drug release kinetic study. Optimization formulation batches showed angle of repose and Hausner's ratio with good flow and packing ability. Friability, hardness, weight variation and drug content of all batches were passed as per pharmacopoeia limits. Invitro drug release study of optimization batches showed an increase in crospovidone and Eudragit L30D concentration resulted in the immediate drug release and increased lag time. The results of in vitro release kinetics indicate that the release from all formulations follows zero order release kinetic model.

The process was optimized for the dependent (responses) variables selected based on criteria of attaining the maximum % drug release after lag time and lag time. ANOVA indicated that assumed regression models were significant and valid for each considered response. It was observed from the response curves and contour plots responses that increasing coating weight gain of Eudragit L30D retard the water uptake and rises lag time significantly. Increasing level of crospovidone creats more pressure over outer Eudragit L30D coat due to its wicking and swelling ability of disintegrant is best utilized and thus releases drug immediately by rupturing the outer membrane. According to the design the best area for formulation to obtain desired responses was found. The linear correlation plots drawn between the predicted and actual values for all the batches of optimization. Thus the low magnitudes of error as well as the values of R2 in the present investigation prove the high prognostic ability of the optimization technique by factorial design. The result shows that the observed responses were inside the constraints and close to predicted responses, and, therefore, factorial design is valid for predicting the optimum formulation.

Response surface methodology (RSM) is a widely practiced approach in the development and optimization of drug delivery devices. Based on the principle of design of experiments, the methodology encompasses the use of various types of experimental designs, generation of polynomial equations and mapping of the response over the experimental domain to determine the optimum formulation(s). The technique requires minimum experimentation and time, thus proving to be far more effective and cost effective than the conventional methods of formulating the dosage forms.

## Conclusions:

We show that the budesonide pH dependent pulsatile burst release tablets are an option for ileo-cecal targeting for achieving the desired lag time. Lag time and target release was observed by good correlation between in vitro and drug release kinetic studies. Thus, the designed formulation is promising for ileo-cecal targeted pulsatile drug delivery system in the management of Crohn's disease.

## Figures and Tables

**Table 1:** The developed Experimental design: factors and responses.

Factors (independent variables)	Levels used			Responses (dependent variables)
	-1	0	1	
X1=(B) Extent of Crospovidone	15	20	25	Y1= % drug release within 90 min. after lag time
X2= (A) Extent of Eudragit	20	25	30	Y2= lag time of 5h
L30D coating weight gain (%)				

**Table 2:** Composition of budesonide experimental formulations (runs)

Batch No.	Extent of Crospovidone (mg)	Extent of Eudragit L30D coating (%w/w)
OF1	25	30
OF2	20	30
OF3	25	25
OF4	20	25
OF5	15	25
OF6	15	30
OF7	20	20
OF8	15	20
OF9	25	20

**Table 3:** Evaluation and characterization of optimize tablet granules (OF1-OF9)

Formulation code	Bulk density gm/ml	Tapped density gm/ml	Carr's index (%)	Hausner's ratio	Angle of repose (°)
OF1	0.740±0.03	0.880±0.04	15.90±0.14	1.18±0.04	24.18±1.52
OF2	0.771±0.03	0.851±0.04	9.40±0.21	1.10±0.03	22.35±1.20
OF3	0.810±0.04	0.875±0.03	7.42±0.05	1.08±0.03	24.11±1.57
OF4	0.824±0.06	0.933±0.05	11.68±0.07	1.13±0.02	23.68±3.53
OF5	0.725±0.06	0.871±0.03	14.80±0.06	1.20±0.05	24.09±2.52
OF6	0.814±0.05	0.952±0.02	14.49±0.09	1.16±0.01	24.12±1.91
OF7	0.707±0.05	0.830±0.02	14.81±0.13	1.17±0.02	23.11±0.97
OF8	0.793±0.03	0.901±0.03	11.98±0.10	1.13±0.01	25.58±1.25
OF9	0.781±0.04	0.861±0.03	9.290.13	1.10±0.03	23.18±3.03
(All value represents mean ± SD (n=3)

**Table 4:** Evaluation and characterization of optimize tablet (OF1-OF9)

Formulation code	Thickness in mm	Diameter in mm	Hardness in Kg/cm2	Friability in % w/w	Weight variation in mg	Drug content (%)
OF1	3.60±0.03	6.02±0.01	7.7±0.07	0.139±0.02	179.20±0.78	99.51±0.03
OF2	3.65±0.02	6.03±0.03	7.5±0.14	0.199±0.03	182.71±1.08	99.31±0.02
OF3	3.62±0.01	6.01±0.03	7.5±0.06	0.182±0.02	176.32±2.21	99.52±0.02
OF4	3.58±0.01	5.99±0.03	7.6±0.04	0.209±0.01	178.05±2.30	99.78±0.03
OF5	3.60±0.02	6.00±0.01	7.8±0.11	0.168±0.03	185.02±2.51	99.73±0.04
OF6	3.58±0.02	6.01±0.02	7.8±0.05	0.139±0.02	183.53±3.65	99.18±0.05
OF7	3.77±0.01	6.02±0.01	8.2±0.04	0.165±0.04	184.78±1.14	99.36±0.04
OF8	3.62±0.03	6.01±0.01	7.9±0.02	0.189±0.03	179.44±2.57	99.40±0.10
OF9	3.62±0.01	6.01±0.03	7.8±0.11	0.182±0.02	182.71±1.08	99.51±0.03
(All value represents mean ± SD (n=3)

**Table 5:** In vitro drug release kinetics of budesonide optimization batches OF1-OF9

Batch code	R2 (coefficient of determination) of various Kinetic Models			
	Zero order	First order	Higuchi release	Korsmeyer and Peppas release
OF1	0.865	0.743	0.787	0.736
OF2	0.57	0.464	0.477	0.642
OF3	0.655	0.524	0.536	0.278
OF4	0.873	0.757	0.799	0.734
OF5	0.564	0.428	0.475	0.405
OF6	0.623	0.492	0.512	0.431
OF7	0.868	0.73	0.792	0.732
OF8	0.487	0.324	0.408	0.412
OF9	0.618	0.51	0.591	0.349

**Table 6:** Experimental runs and observed results for OF1-OF9

Std	Run	Factor X1 : A	Factor X2: B	Response Y1: 90% drug release	Response Y2: Lag time (hr)
2	1	25	30	84.36	4.5
3	2	20	30	85.17	5
9	3	25	25	93.44	4.5
8	4	20	25	94.13	5
5	5	15	25	90.83	5
6	6	15	30	89.34	5
4	7	20	20	91.04	4
1	8	15	20	92.71	5
7	9	25	20	83.48	4

**Table 7:** Analysis of Variance for response 90% drug release after lag time

Source	Sum of Squares	df	Mean Square	F Value	p-value Prob>F	
Model	93.31	5	18.66	1.42	0.041	Significant
A	22.43	1	22.43	1.71	0.0825	
B	11.65	1	11.65	0.89	0.0159	
AB	4.52	1	4.52	0.34	0.0989	
A2	2.36	1	2.36	0.18	0.0002	
B2	52.36	1	52.36	3.99	0.1398	
Residual	39.42	3	13.14			
Cor total	132.73	8				

**Table 8:** Analysis of Variance for lag time

Source	Sum of Squares	df	Mean Square	F Value	p-value Prob>F	
Model	1.04	2	0.52	6.82	0.0285	Significant
A	0.67	1	0.67	8.73	0.0255	
B	0.38	1	0.38	4.91	0.0686	
Residual	0.46	6	0.076			
Cor total	1.5	8				

**Table 9:** Predicted and actual response of budesonide optimization batches (OF1-OF9)

Responses	Run order	Predicted Value	Actual Value	Prediction Error* (%)
90% drug release after lag time	1	85.06	84.36	0.82
	2	87.01	85.17	2.11
	3	90.5	93.44	3.24
	4	93.52	94.13	0.65
	5	94.37	90.83	3.75
	6	86.8	89.34	2.92
	7	89.8	91.04	1.38
	8	91.71	92.71	1.16
	9	85.72	83.48	2.61
Lag time	1	4.58	4.5	1.81
(hr)	2	4.92	5	1.68
	3	4.33	4.5	3.92
	4	4.67	5	7.06
	5	5	5	0
	6	5.25	5	4.76
	7	4.42	4	9.5
	8	4.75	5	5.26
	9	4.08	4	2.03
